# Light from Decay: Chemiluminescence as a Kinetic Fingerprint of Dammar Resin Oxidation

**DOI:** 10.3390/molecules31091443

**Published:** 2026-04-27

**Authors:** Andreas Buder

**Affiliations:** Research Institute Materiality in Art and Culture, Bern Academy of the Arts, Bern University of Applied Sciences, Fellerstrasse 11, 3027 Bern, Switzerland; andreas.buder@hkb.bfh.ch

**Keywords:** chemiluminescence, thermo-oxidative degradation, isoconversional kinetics, hydroperoxides, stabilizers, conservation science, natural resins, varnish aging

## Abstract

In this study, chemiluminescence (CL) is presented as a highly sensitive, mechanistically coupled method for investigating the thermo-oxidative aging of dammar resin, a triterpenoid natural resin of central relevance to conservation science. In contrast to conventional spectroscopic techniques, CL does not primarily reflect the accumulated oxidation state; instead, it selectively detects the formation and decomposition of reactive peroxide and hydroperoxide intermediates, thereby providing an early view of the oxidative reactivity of the material. Measurements performed under inert and oxidative atmospheres provide a clear distinction between pre-existing oxidative damage and ongoing autoxidation. Correlation with Fourier-transform infrared (FTIR) spectroscopy demonstrates that oxidized functional groups are not necessarily associated with high oxidative reactivity, underscoring the functional advantage of chemiluminescence for stability assessment. The combination of dynamic CL measurements with model-free isoconversional kinetics has been shown to reveal the pronounced dependence of effective activation energy on the extent of the reaction. This α-dependence confirms the multistep nature of dammar oxidation and highlights the limitations of classical Arrhenius models. Furthermore, chemiluminescence is an effective screening tool for evaluating stabilizers and synergistic additive combinations, providing a robust basis for kinetic modeling and evidence-based decision-making in conservation science.

## 1. Introduction

### 1.1. Dammar Resin in Conservation Practice

Natural triterpenoid resins, particularly dammar resin, have played a central role in the history and practice of painting conservation. Since the nineteenth century, dammar has been extensively utilized as a picture varnish, a practice primarily attributable to its advantageous film-forming properties, elevated optical transparency, and comparatively minimal intrinsic coloration. The substance’s optical effects include enhanced gloss, color saturation, and depth perception, and these have been identified as key factors in its ongoing utilization, despite the presence of well-documented aging issues [1,2].

Even under moderate environmental conditions, dammar undergoes progressive aging, typically yellowing, embrittlement, increased crosslinking, and a gradual loss of solubility. These changes not only compromise the aesthetic perception of historical paintings but also confront conservation practice with the recurring need for invasive varnish removal, which entails inherent risks for the underlying paint layers. Therefore, a detailed understanding of the underlying aging mechanisms is a prerequisite for the evidence-based assessment of existing varnishes and the development of more stable formulations and conservation strategies [3,4,5,6,7].

### 1.2. Oxidative Aging as the Dominant Degradation Mechanism

The aging of dammar resin is governed predominantly by auto-oxidative processes. In the presence of oxygen, hydroperoxides form as key reactive intermediates, whose thermal or catalytic decomposition initiates cascades of radical chain reactions. These processes form polar carbonyl and hydroxyl functionalities, secondary crosslinking reactions, and chromophoric structures responsible for the characteristic yellowing of aged dammar films [3,7,8,9].

Numerous analytical studies have demonstrated that these processes are spatially and chemically heterogeneous. Natural resin lumps and historical varnish layers exhibit pronounced gradients in oxidation and crosslinking: surface-near regions are typically far more pre-oxidized than the material in the interior. This heterogeneity directly affects optical, mechanical, and solubility-related properties and represents a major challenge for representative analytical characterization [3,9,10,11].

### 1.3. Limitations of Classical Thermoanalytical Methods

In materials science and conservation research, thermoanalytical techniques such as differential scanning calorimetry (DSC) and thermogravimetry (TG) have long been employed to investigate aging processes. Although these methods provide robust and standardized parameters, they reach fundamental limits when used to characterize early oxidative stages. The detection of reactive intermediates—particularly hydroperoxides—often requires elevated temperature regimes, where secondary reactions, signal overlap, and experimental artefacts are difficult to avoid [5,11,12].

From a kinetic perspective, it has also become evident that complex solid-state reactions, such as the oxidative aging of natural resins, cannot be adequately described by simple Arrhenius models with constant parameters. Effective activation energies and reaction rates depend on the extent of conversion, local material properties, and the thermal history of the sample. This intrinsic multistep behavior frequently remains obscured in classical, model-dependent kinetic evaluations [13,14,15,16].

### 1.4. Chemiluminescence as a Mechanistically Coupled Tool

Against this background, chemiluminescence (CL) has emerged as a particularly powerful method for investigating oxidative degradation processes. CL is based on the emission of weak light signals generated during the decomposition of high-energy oxidative intermediates, most notably peroxide and hydroperoxide species. Crucially, the emitted signal is directly coupled to oxidative reaction mechanisms rather than reflecting purely thermophysical effects [11,17,18].

A key advantage of chemiluminescence is its high sensitivity, allowing oxidative processes to be detected at temperatures well below those required by conventional thermoanalytical techniques. By varying the measurement atmosphere, CL can distinguish between ongoing oxidation under oxygen and the decomposition of pre-accumulated oxidative intermediates under inert conditions. This provides direct access to the oxidative reactivity of a material rather than its accumulated oxidation state.

Chemiluminescence has been successfully applied for several decades in polymer and materials research, including studies of early stages of oxidation, quantification of peroxide populations, and the screening of stabilizing additives. More recent developments—such as imaging CL techniques and integration with model-free kinetic approaches—have further expanded its analytical potential [11,19,20,21]. In the context of conservation science, these capabilities make CL particularly well suited for mechanistic and kinetic investigations of resin aging processes.

### 1.5. Aim and Structure of This Review

This review systematically assesses the potential of chemiluminescence for the conservation and scientific investigation of dammar resin. Chemiluminescence is not considered a standalone technique but rather part of an integrated analytical framework that combines mechanistic, kinetic, and thermodynamic information.

The review is structured as follows: First, the chemical structure and oxidative reactivity of dammar resin are summarized. Subsequently, the principles, measurement strategies, and interpretative possibilities of chemiluminescence in organic solid-state systems are discussed. Particular emphasis is placed on correlating CL signals with complementary methods such as Fourier-transform infrared (FTIR) spectroscopy to support mechanistic interpretation. On this basis, model-free isoconversional kinetic approaches and their thermodynamic embedding are introduced. Finally, selected applications relevant to conservation practice are discussed, and perspectives are outlined for integrating CL-derived parameters into advanced degradation models, including data-driven ones.

## 2. Dammar Resin as a Model System for Oxidative Degradation in Conservation

### 2.1. Origin, Composition, and Material-Relevant Properties

Dammar resin is a natural triterpenoid resin obtained from various tree species of the *Dipterocarpaceae* family. The raw material occurs as resinous lumps that, after drying, are readily soluble in non-polar to weakly polar organic solvents. This property is the basis for its historical and continued use as a varnish material in painting conservation [5,9].

**Figure 1:**
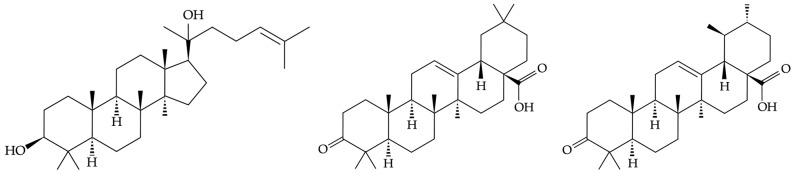
Dammarendiol (**left**), oleanonic acid (**center**), and ursonic acid (**right**) are the major constituents of dammar, with the dammarane skeleton featured [11].

The major components of dammar are dammarane-type triterpenes; in addition, compounds with oleanane-, ursane-, and hopane-type skeletons have been identified (Figure 1). Dammar further contains complex mixtures of resin acids, resin alcohols, esters, ketones, and strongly unsaturated compounds such as β-resene [8,9,22,23].

From a materials perspective, this composition gives rise to several properties of particular relevance for conservation: good film formation at room temperature, high optical transparency immediately after application, and comparatively low initial coloration. At the same time, unsaturated structural motifs render the material highly susceptible to oxidative degradation [3,4,8].

### 2.2. Autoxidation of Dammar Resin and Chemiluminescent Reaction Pathways

The aging of dammar resin is dominated by auto-oxidative processes, as commonly observed in unsaturated organic solids. These processes originate from numerous reactive structural motifs within the triterpenoid resin mixture, particularly isolated and conjugated double bonds, as well as functional groups prone to hydrogen abstraction. Even under moderate environmental conditions, molecular oxygen diffuses into the resin matrix and reacts with these sites, initiating radical chain reactions [9,10].

In principle, the autoxidation of dammar follows the classical mechanism of solid-state oxidation, comprising initiation, propagation, and termination. After the formation of initial alkyl radicals, rapid reaction with oxygen yields peroxyl radicals (ROO•). These species constitute key intermediates, as they both propagate the chain reaction via hydrogen abstraction and participate in various termination and decomposition reactions [9,11].

A characteristic feature of dammar oxidation is the accumulation of hydroperoxides (ROOH). These compounds are thermodynamically metastable, can persist over extended periods, and act as reservoirs of chemical energy. Their decomposition—thermal, photochemical, or catalyzed—forms highly reactive alkoxy and hydroxyl radicals, which trigger further oxidation, rearrangement, and crosslinking reactions. The development of polar carbonyl and hydroxyl functionalities, increased crosslink density, and the formation of chromophoric structures can be directly attributed to these processes, providing a mechanistic explanation for macroscopic aging phenomena such as yellowing, embrittlement, and reduced solubility [3,9,11].

The chemiluminescence observable during the aging of dammar does not originate from the initiation or propagation stages of oxidation; rather, it comes predominantly from the highly exothermic termination and decomposition reactions of oxidative intermediates. The most important mechanistic contribution to light emission arises from the bimolecular recombination of peroxyl radicals according to the Russell mechanism, shown in Equation (1). In this process, two ROO• radicals react via a short-lived tetraoxide intermediate, which decomposes with the release of molecular oxygen and the formation of an electronically excited carbonyl species. Relaxation of this excited state results in photon emission [11,19,24,25]:

2 ROO• → ROOOOR → R = O* + ROH + O_2_ (1)

In addition, the thermal decomposition of hydroperoxides makes a significant contribution to chemiluminescence, particularly under inert atmospheres. In this case, alkoxy and hydroxyl radicals are generated, whose subsequent reactions—such as cage recombination or β-scission—may likewise lead to the formation of electronically excited carbonyl species. Crucially, all of these emission processes are strictly linked to the presence of reactive oxidative intermediates [11,19,26,27].

A characteristic feature of dammar resin is that even the untreated starting material contains a substantial fraction of oxidative precursors. Therefore, pronounced chemiluminescence signals can be detected even in the absence of additional oxygen supply, demonstrating that pre-existing hydroperoxides and peroxyl species represent a major source of the CL signal. Under oxidative atmospheres, chemiluminescence results from the interplay between ongoing peroxide formation and its energetic termination.

Accordingly, chemiluminescence in dammar is primarily an indicator of instantaneous oxidative reactivity. It selectively probes those reactions that are decisive for further aging while not directly reflecting the accumulation of stable early or late oxidation products. This selectivity renders CL particularly well suited for investigating early aging stages, analyzing kinetic transitions, and assessing the effectiveness of stabilizers [11].

### 2.3. Spatial Heterogeneity and Aging Gradients

A characteristic feature of dammar resin is the pronounced spatial heterogeneity of its aging behavior. Even in the raw material, distinct oxidation gradients are present: outer regions of resin lumps are significantly more pre-oxidized than the interior as a result of prolonged exposure to atmospheric oxygen and light. These gradients persist after processing and can likewise be observed in dried varnish films [11,28].

Consequently, the chemical state of dammar is far from homogeneous and varies strongly on a local scale. Surface-near zones exhibit higher concentrations of oxidation products, radicals, and peroxides, whereas deeper regions remain comparatively less altered. This inhomogeneity affects not only optical properties but also mechanical behavior and reaction kinetics.

For analytical investigations, this poses a fundamental challenge: point-based measurements or techniques with limited penetration depth often capture only partial aspects of the material. Therefore, methods capable of detecting even low concentrations of reactive intermediates while simultaneously discriminating between different oxidation states provide particular analytical value.

### 2.4. Dammar as an Appropriate Reference and Model System

Owing to these characteristics, dammar resin represents an exceptionally suitable model system for studying oxidative degradation processes in conservation science. Several aspects are decisive in this regard. First, its chemical aging behavior is well documented and has been investigated using a broad range of complementary analytical techniques, allowing new methods to be embedded within an established reference framework. Second, the oxidation proceeds sufficiently slowly to render early reaction stages experimentally accessible. Third, the material is of direct relevance to conservation practice due to its historical and ongoing use [11].

Within the context of this review, dammar thus serves not merely as an object of investigation but as an exemplary system that clearly demonstrates the strengths of chemiluminescence: the detection of low-concentration oxidative intermediates; the distinction between oxidation state and oxidation propensity; and the kinetic description of complex, multi-step solid-state reactions [11].

## 3. Correlation Between Chemiluminescence and FTIR Spectroscopy

**Figure 2:**
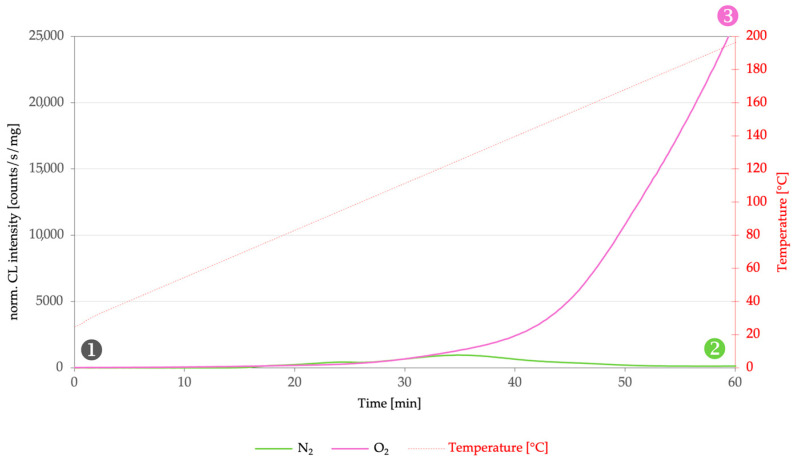
Chemiluminescence (CL) intensities of dammar resin (inner material, unstabilized) measured under N_2_ (green) and O_2_ atmosphere (purple). Sample 1 corresponds to the reference material, Sample 2 to the specimen after CL measurement under inert atmosphere (N_2_), and Sample 3 to the specimen subsequently analyzed by FTIR after CL measurement under oxidative atmosphere (O_2_). Chemiluminescence measurements were performed using an ACL-Instruments system with a gating time of 1000 ms and a constant gas flow of 60 mL min^−1^; samples with a mass of 5.5 mg consisting of defined, mechanically fragmented, and homogenized dammar resin material were heated from 30 °C to 200 °C within 60 min, corresponding to a heating rate of 2.83 K min^−1^.

**Figure 3:**
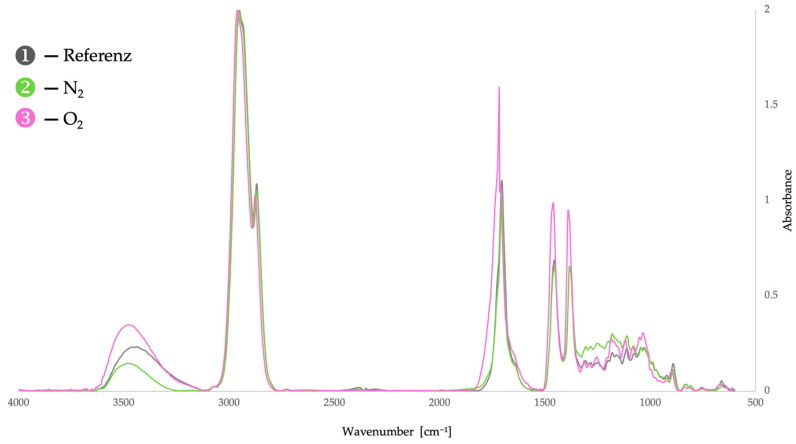
FTIR spectra of dammar resin samples recorded at characteristic CL measurement points (reference, N_2_, O_2_), acquired using a LUMOS II FT-IR microscope (Bruker) operated in transmission mode (4000–500 cm^−1^, 64 co-added scans, TE-MCT detector). The spectra were baseline-corrected and normalized.

The combined application of chemiluminescence (CL) and Fourier-transform infrared (FTIR) spectroscopy enables a clear distinction between oxidation state and oxidative reactivity in dammar resin [6,9,29]. This complementarity is particularly evident when comparing the three investigated experimental conditions (Figure 2 and Figure 3): pre-oxidized reference material (gray), samples after CL measurement under an inert atmosphere (green, N_2_), and samples after CL measurement under an oxidative atmosphere (purple, O_2_).

### 3.1. Reference: Pre-Oxidized Starting Material

FTIR spectra of untreated dammar resin (Figure 3) exhibit pronounced absorption bands in the O–H, C=O, and C–O regions, confirming that the material is already oxidatively pre-damaged prior to any experimental treatment [9]. These spectral features reflect the presence of hydroxyl, carbonyl, and ether or ester functionalities formed during the harvesting, storage, and handling of the resin.

At the same time, measurable chemiluminescence can be observed from the reference material (Figure 2), even under an inert atmosphere. This finding indicates that a fraction of the oxidized functional groups present is energetically metastable and capable of undergoing exothermic decomposition reactions. While FTIR documents the existing oxidation state of the material, CL provides direct evidence of the residual oxidative reactivity associated with this pre-oxidation. Even at this stage, it becomes apparent that FTIR alone does not allow reliable conclusions to be drawn regarding the future oxidation potential of the material [11,21].

### 3.2. After CL Measurement Under Inert Atmosphere (N_2_)

After CL measurements performed under nitrogen, FTIR spectroscopy reveals a decrease in those oxidation-related functional groups that previously correlated with CL emission, most notably in the carbonyl and C–O regions (Figure 3). Under inert conditions, pre-existing peroxide and hydroperoxide species are thermally consumed without the formation of new oxidative intermediates.

This situation is of particular methodological significance:

- Chemiluminescence selectively records the consumption of reactive oxidative intermediates.
- FTIR independently confirms this process as a decrease in stable functional groups.

Thus, the combined analysis makes experimentally visible what FTIR alone cannot resolve: the identification of those oxidation products that remain chemically reactive. The results clearly demonstrate that oxidation state and oxidation dynamics are not equivalent quantities [9,21].

### 3.3. After CL Measurement Under Oxidative Atmosphere (O_2_)

Following CL measurements under oxygen (Figure 2), the situation is reversed. FTIR spectra now show an increase in oxidation-related absorption bands in the C=O and C–O regions (Figure 3) and pronounced chemiluminescence can be observed simultaneously. Under oxidative conditions, propagation and termination reactions proceed in parallel: new peroxides are formed, while others decompose and contribute to CL emission.

These findings confirm that exposure to oxygen—and, in practice, light-assisted oxygen availability—markedly accelerates oxidative aging, yet not in a manner proportional to the overall radical concentration. Chemiluminescence proves to be a particularly sensitive indicator of these dynamic processes, which are only indirectly and cumulatively reflected in FTIR spectra [9,11].

### 3.4. Methodological Implications of Chemiluminescence

Direct comparison highlights the key methodological advantage of CL. Whereas FTIR spectroscopy captures the accumulated oxidation state and reaches analytical limits when differences are subtle—owing to baseline effects, spectral overlap, and sample inhomogeneity—CL selectively probes the reactivity of oxidative intermediates.

The three experimental conditions demonstrate the following:

- FTIR alone cannot determine whether oxidized functional groups remain relevant for further aging.
- Chemiluminescence enables this distinction by selectively detecting termination and decomposition reactions.
- In combination, CL and FTIR confirm the fundamental aging mechanisms of dammar resin while extending their interpretation by a distinct kinetic and functional perspective [3,9].

## 4. Chemiluminescence as a Tool for Assessing Stabilization and Reactivity

### 4.1. Stabilization Concepts for Dammar Resin

The limited intrinsic stability of dammar resin against thermo-oxidative aging has repeatedly motivated the development and testing of stabilizing additives in conservation research. The underlying concepts can be broadly assigned to two principal mechanisms: on the one hand, reduced or retarded radical formation, for example, through light-protective additives; on the other hand, the interception of previously formed reactive intermediates by antioxidants [4,30,31].

However, as shown by previous studies, the experimental assessment of such stabilization strategies is methodologically demanding. Classical spectroscopic techniques such as FTIR predominantly record stable oxidation products and respond only weakly to differences in oxidative reactivity. Especially at moderate oxidation levels, changes in FTIR spectra often remain subtle or become clearly discernible only after prolonged artificial aging. This limitation is particularly relevant for pre-oxidized starting materials, as is characteristic of dammar resin [16,32].

### 4.2. Chemiluminescence as a Sensitive Measure of Stabilizer Performance

Chemiluminescence offers a decisive methodological advantage over these techniques, as it is selectively sensitive to oxidative intermediates that actively contribute to the progression of aging. Consequently, the effectiveness of stabilizers is not evaluated via the global oxidation state but through changes in the oxidative reactivity of the material.

**Figure 4:**
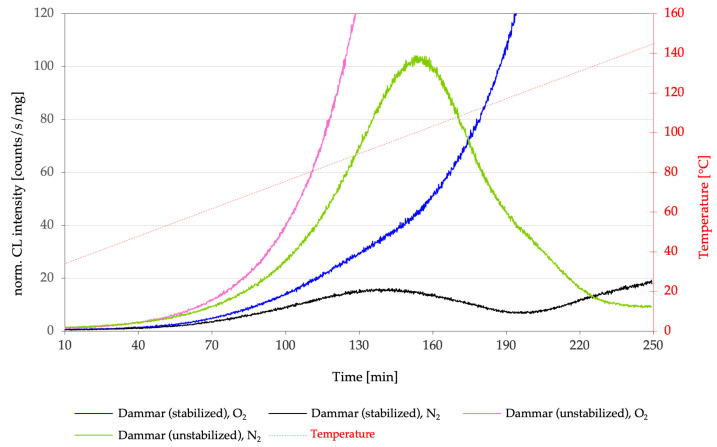
The figure presents the CL intensities of unstabilized dammar (green and purple) and stabilized dammar (black and blue) for a dammar resin sample from the inner region, measured under N_2_/O_2_ atmospheres.

In CL measurements (Figure 4), effective stabilization—when comparing untreated and stabilized films—is reflected by characteristic features:

- A pronounced extension of the oxidation induction time;
- Reduced emission intensities;
- A shift in CL maxima toward higher temperatures.

These parameters enable a direct functional evaluation of stabilization effects, well before stable oxidation end products accumulate to a detectable extent [11].

### 4.3. Individual Stabilizers

Phenolic antioxidants (e.g., Irganox^®^-type additives) significantly reduce CL emission intensity, accompanied by a pronounced extension of the oxidation induction period. This behavior is consistent with their role as radical scavengers within the auto-oxidative chain reaction. Under oxidative conditions, their effect is particularly evident: the onset temperatures of CL shift markedly to higher values (Figure 4), indicating an effective delay in the formation of reactive peroxide species [4,11].

Hindered amine light stabilizers (HALS), such as Tinuvin^®^ 292, exhibit an even stronger stabilizing effect. Their efficacy does not rely solely on scavenging individual radicals but rather on a cyclic reaction mechanism (Denisov cycle), interrupting radical chain reactions over extended periods. In CL curves of stabilized dammar films, this manifests as strongly reduced emission intensities and CL maxima shifted to substantially higher temperatures [4,11,30,33,34].

### 4.4. Synergistic Stabilizer Combinations

Synergistic stabilizer systems have proven to be particularly effective. The combined application of a UV absorber (Tinuvin^®^ 328) with a HALS additive (Tinuvin^®^ 292) results in a pronounced improvement in oxidative stability. The corresponding CL data show

- Very low emission rates across a broad temperature range;
- Markedly extended induction phases;
- Emission maxima shifted to significantly higher temperatures.

This synergistic effect clearly surpasses the performance of all individual stabilizers investigated and, based on both the literature and experimental evidence, is regarded as one of the most effective formulations for stabilizing dammar resin films [4,11,30,33].

Here, the advantage of CL becomes particularly evident: whereas FTIR measurements mainly integrate stable oxidation products, CL primarily records the consumption or suppression of metastable peroxide and hydroperoxide species. Thus, it can reveal whether an additive actively interferes with the radical chain reaction or merely affects the accumulated oxidation state. Differences between stabilization strategies in FTIR spectra that are barely discernible, or not discernible at all, become clearly distinguishable in CL measurements [11].

### 4.5. Relationship Between Stabilization, Reactivity, and Kinetics

A central outcome of CL-based investigations is that stable materials are not necessarily characterized by a low oxidation level but rather by the low reactivity of the remaining oxidized groups. Accordingly, stabilizers can be highly effective even when carbonyl or C–O functionalities remain detectable by FTIR spectroscopy.

This observation is consistent with studies demonstrating that oxidation progress and radical concentration—especially at advanced stages of aging—are no longer proportional. CL renders this non-linear relationship experimentally accessible, thereby enabling a kinetically and functionally grounded assessment of stabilization effects [3,9,11].

## 5. Summary and Outlook

### 5.1. Summary of the Main Findings

The present work demonstrates that chemiluminescence (CL) constitutes a highly sensitive and selective tool for investigating the thermo-oxidative aging of dammar resin. In contrast to conventional spectroscopic methods, CL does not primarily reflect the accumulated oxidation state but instead detects reactive oxidative intermediates—most notably peroxide and hydroperoxide species—that govern the further progression of aging processes [11].

By systematically comparing measurements conducted under inert and oxidative atmospheres, a clear distinction between pre-existing oxidative damage and ongoing oxidation can be established. In combination with FTIR spectroscopy, oxidized functional groups are not necessarily associated with high oxidative reactivity. The results thus confirm earlier findings on the non-linear relationship between oxidation level and aging dynamics while extending these insights through a functional and kinetic perspective [9,11].

A particular methodological strength of chemiluminescence lies in its suitability for evaluating stabilizers. Antioxidants, HALS additives, and UV absorbers induce characteristic changes in CL induction times, emission intensities, and peak temperatures. Synergistic combinations—especially those combining UV absorbers with HALS—prove to be particularly effective and can be clearly differentiated by CL, even when FTIR spectra show only minor differences [4,5,30,31,33].

### 5.2. Model-Free Kinetics: Significance of the α-Dependent Activation Energy

A central insight of this study arises from the application of model-free isoconversional kinetic methods to CL data. Determination of the effective activation energy as a function of the extent of conversion, *E*ₐ(α), demonstrates that the thermo-oxidative degradation of dammar resin cannot be described by a single, time-independent mechanism.

**Figure 5:**
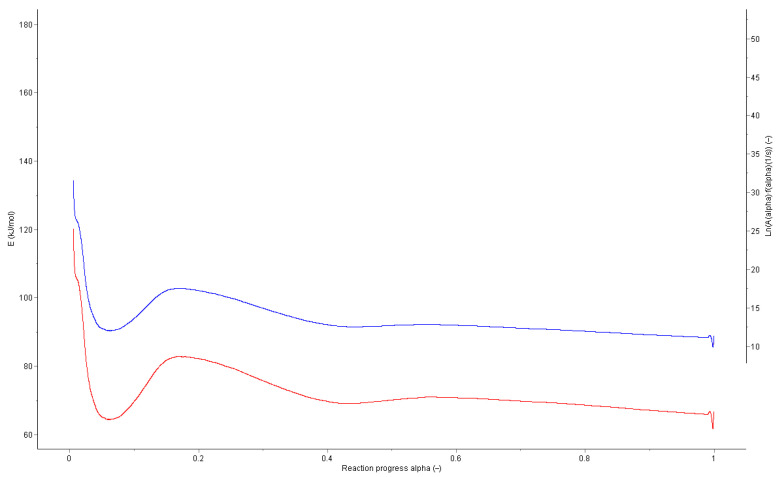
Activation energy (red) and pre-exponential factor (blue) as a function of reaction progress, shown as a Friedman plot for a dammar resin sample from the inner region measured under N_2_/O_2_ atmosphere. The parameters were determined using AKTS-Thermokinetic software (Vers. 4.15) based on a series of non-isothermal chemiluminescence measurements carried out at different heating rates employing a model-free isoconversional approach [11].

Figure 5 illustrates the variation in activation energy over the reaction course. The pronounced change in *E*ₐ with increasing conversion reflects transitions between different rate-determining steps, such as the formation, decomposition, and recombination of hydroperoxides. This α-dependence confirms the multi-step nature of the aging process and highlights the fundamental limitations of classical Arrhenius models based on fixed kinetic parameters [14,15,16,35].

These findings are consistent with recent theoretical approaches demonstrating that deviations from Arrhenius- and Eyring-type behavior are intrinsic to complex, multi-step solid-state reactions and that mechanistic interpretations based on constant activation energies are inherently limited. Concepts such as the transitivity function further emphasize that variable activation energies should not be regarded as empirical artifacts but rather as kinetic fingerprints of the reaction pathway. This renders model-free, CL-based kinetic approaches particularly suitable for describing oxidative aging processes [13,36].

This insight is of particular importance for heterogeneous solid-state systems such as dammar resin. Model-free kinetics allows aging reactions to be described without presupposing a reaction model and enables a more realistic representation of actual material conditions. The kinetic parameters derived from CL measurements can subsequently be applied in isothermal and non-isothermal simulations to estimate time-dependent aging scenarios under defined temperature profiles [14].

### 5.3. Outlook: Linking Chemiluminescence to Data-Driven and AI-Based Approaches

Beyond its immediate analytical application, the concepts employed in this work point toward broader perspectives for data-driven modeling approaches aimed at describing material-related aging processes. In particular, the kinetic parameters derived from chemiluminescence measurements using a model-free approach—most notably α-dependent activation energy—represent physically interpretable quantities that are, in principle, suitable for further mathematical and statistical analysis.

In future studies, such parameters could be used, for example, to classify different levels of material stability, to estimate service lifetimes, or to comparatively evaluate stabilizer formulations. The potential integration of these physically grounded descriptors into data-driven or AI-based modeling approaches may also be possible, with the aim of systematically capturing complex relationships between material conditions, oxidative reactivity, and external aging factors.

Notably, no such AI-based models were developed or tested within the scope of the present work. Rather, this outlook highlights the potential of CL to provide kinetically meaningful, physically justified descriptors that may constitute a robust foundation for future, carefully validated modeling strategies [37,38,39].

### 5.4. Concluding Remarks

In summary, this work demonstrates that CL extends well beyond the role of a sensitive oxidation marker. When combined with model-free kinetic analysis, it enables a dynamic and mechanistically grounded description of the thermo-oxidative aging of dammar resin. This approach allows for a differentiated assessment of oxidative stability, a reliable evaluation of stabilization strategies, and a realistic estimation of future aging behavior.

As a key analytical tool, CL is highly relevant not only to conservation science but also to materials science, polymer science, and the broader field of research on the aging of materials. However, CL does not capture the entire accumulated oxidation state of a material and does not provide structurally specific identification of individual aging products. Its particular strength lies instead in selectively detecting the oxidative reactivity of energetically relevant intermediates, which—when combined with structure-elucidating methods—enables a differentiated and robust understanding of complex aging processes.

## Data Availability

No new data were created or analyzed in this study.
